# SARS-CoV-2 Proteome-Wide Analysis Revealed Significant Epitope Signatures in COVID-19 Patients

**DOI:** 10.3389/fimmu.2021.629185

**Published:** 2021-03-23

**Authors:** Tatjana Schwarz, Kirsten Heiss, Yuvaraj Mahendran, Fiordiligie Casilag, Florian Kurth, Leif E. Sander, Clemens-Martin Wendtner, Manuela A. Hoechstetter, Marcel A. Müller, Renate Sekul, Christian Drosten, Volker Stadler, Victor M. Corman

**Affiliations:** ^1^ Institute of Virology, Charité—Universitätsmedizin Berlin, Corporate Member of Freie Universität Berlin, Humboldt-Universität zu Berlin, and Berlin Institute of Health, Berlin, Germany; ^2^ PEPperPRINT GmbH, R&D unit, Heidelberg, Germany; ^3^ Department of Infectious Diseases and Respiratory Medicine, Charité—Universitätsmedizin Berlin, Corporate Member of Freie Universität Berlin, Humboldt-Universität zu Berlin, and Berlin Institute of Health, Berlin, Germany; ^4^ Munich Clinic Schwabing, Academic Teaching Hospital, Ludwig-Maximilians University (LMU), Munich, Germany; ^5^ German Centre for Infection Research, Associated Partner Charité, Berlin, Germany

**Keywords:** antibody response, epitope identification, B cells, diagnostic, respiratory tract infection

## Abstract

The WHO declared the COVID-19 outbreak a public health emergency of international concern. The causative agent of this acute respiratory disease is a newly emerged coronavirus, named SARS-CoV-2, which originated in China in late 2019. Exposure to SARS−CoV−2 leads to multifaceted disease outcomes from asymptomatic infection to severe pneumonia, acute respiratory distress and potentially death. Understanding the host immune response is crucial for the development of interventional strategies. Humoral responses play an important role in defending viral infections and are therefore of particular interest. With the aim to resolve SARS-CoV-2-specific humoral immune responses at the epitope level, we screened clinically well-characterized sera from COVID-19 patients with mild and severe disease outcome using high-density peptide microarrays covering the entire proteome of SARS-CoV-2. Moreover, we determined the longevity of epitope-specific antibody responses in a longitudinal approach. Here we present IgG and IgA-specific epitope signatures from COVID-19 patients, which may serve as discriminating prognostic or predictive markers for disease outcome and/or could be relevant for intervention strategies.

## Introduction

COVID-19 is an acute respiratory disease caused by SARS-CoV-2, which emerged in China in December 2019 (1, 2). Due to the rapid global spread and increase in number of cases, the World Health Organization (WHO) declared COVID-19 a pandemic in March 2020. As of November 13, 2020, more than 51 million confirmed COVID-19 cases have been reported from 220 countries, areas, or territories with over 1.2 million fatalities (3). The course of disease ranges from asymptomatic to milder symptoms such as fever and cough, to severe outcomes with pneumonia, respiratory distress, and potentially death (4–6). Most vulnerable for severity are elderly people and individuals with comorbidities, such as obesity. Enormous efforts are ongoing, aiming to develop efficacious and timely drugs, and 48 vaccines are currently in clinical evaluation and the first already in use (7, 8). The rapid availability of sequence data enabled the development of molecular diagnostic tests for the detection of SARS-CoV-2 (9, 10) which are key for patient management and the implementation of measures to combat the pandemic. Intensive research is ongoing to develop and validate specific and sensitive serological assays (11–23), mainly focusing on IgG, IgM, and/or IgA antibody response against single target proteins. However, these assays reflect only a small fraction of the humoral response. Furthermore, possible antibody cross-reactivity due to sequence similarities between SARS-CoV-2 and the four endemic human coronaviruses, and especially, an even higher degree of similarity to SARS-CoV is a challenge to overcome (14, 18, 19). In-depth understanding of SARS-CoV-2 specific antibody responses is not only crucial for the development of diagnostics but also for epidemiological studies and intervention strategies, such as vaccine development and monitoring. To date, proteome-wide analyses of humoral responses elicited in COVID-19 patients are still limited (24–27). Microarray-based technologies are ideally suited for profiling proteome-wideantibody responses in a high-throughput context.

In this study, we present a proteome-wide analysis on epitope level SARS-CoV-2 specific antibody responses using peptide microarrays. The high peptide-to-peptide overlap of our SARS-CoV-2 proteome array allows a high-resolution epitope analysis giving a detailed picture of antibody binding patterns, contributing to better characterization of SARS-CoV-2-specific humoral immune responses.

## Material and Methods

### Serum Samples/Study Population

For longitudinal analysis and comparison of the humoral response, sera of PCR-confirmed COVID-19 patients with mild (n=9) and severe (n=7) course of disease were used. Patients with mild courses are part of a well-characterized cohort (28, 29). Patients with severe courses, defined by the need of admission to an intensive care unit, are included in the Pa-COVID-19 study at Charité - Universitätsmedizin Berlin (30). Serum samples (n=7) for SARS-CoV-2 naive control group were collected from healthy volunteers with no contact to COVID-19 patients and no reported COVID-19 associated symptoms. Ethical approval was granted by the local Ethics Committee of the Charité - Universitätsmedizin Berlin (EA2/066/20, EA1/068/20) and the Ethics Committee at the Medical Faculty of the Ludwig Maximilians Universität Munich (vote 20-225 KB) in accordance with the guidelines of the Declaration of Helsinki.

### ELISA

For the detection of SARS-CoV-2 specific antibodies to the spike (S) protein and to the Nucleocapsid (NCP) protein, we used anti-SARS-CoV-2 S1 IgG, anti-SARS-CoV-2 S1 IgA and anti−SARS-CoV-2 NCP ELISAs according to manufacturer’s instructions (EUROIMMUN Medizinische Labordiagnostika AG, https://www.euroimmun.com). Serum samples were tested at a 1:101 dilution using the fully EUROIMMUN Analyzer I. For all analyses, optical density (OD) was detected at 450 nm and ratios were calculated by dividing the observed OD by that of the calibrator included in the kit. The OD ratio can be utilized as a relative measure for the concentration of antibodies in serum. For IgG and IgA, an OD ratio of 0.8-1.09 was considered borderline, and values above 1.1 to be reactive.

### Plaque Reduction Neutralization Test

To test neutralizing activity of SARS-CoV-2 antibodies of ELISA reactive sera plaque reduction neutralization test (PRNT) were done as previously described (29, 31). Briefly, Vero E6 cells were seeded in 24-well-plates and incubated overnight. Heat-inactivated sera were diluted in OptiPro and mixed 1:1 with 100 plaque forming units of SARS-CoV-2 (strain MT270112.01). Each well was incubated with the serum-virus solutions for 1 hour at 37°C. Then the supernatants were discarded, cells were washed with PBS, and overlaid with 1.2% Avicel solution in DMEM. After 3 days at 37°C, cells were fixed and inactivated by utilizing a 6% formaldehyde/PBS solution and stained with crystal violet. Serum dilutions with a plaque reduction of 50% (PRNT50) are referred to as titers.

### Peptide Microarrays

The PEPperCHIP^®^ SARS-CoV-2 Proteome Microarray (PEPperPRINT GmbH, Germany) covers the entire proteome of SARS-COV-2 isolate Wuhan-Hu-1 (GenBank ID: MN908947.3). The protein sequences of ORF1a/b, Spike protein, ORF3a, Envelope protein, Membrane glycoprotein, ORF6, ORF7a, ORF8, Nucleocapsid phosphoprotein and ORF10 were translated into 15 amino acid peptides with a peptide-peptide overlap of 13 amino acids. This results in 4,883 individual peptides, which were printed in duplicates. The PEPperCHIP^®^ SARS-CoV-2 Proteome Microarray further contains influenza hemagglutinin and polio control peptides (108 spots each control peptide).

The peptide microarrays were incubated for 15 minutes in phosphate buffered saline supplemented with 0.05% Tween 20 (PBS-T, pH 7.4) and blocked for 30 minutes with Rockland Blocking Buffer (RL) (Rockland Immunochemicals) at room temperature. Prior to immunoassay, patient sera were heat-inactivated at 56°C for 30 minutes. Microarrays were incubated at serum dilutions of 1:100 in 10% RL/PBS-T overnight at 4°C with orbital shaking. After washing (3 times with PBS-T for 1 minute), peptide binding was detected with isotype-specific secondary goat anti-human IgG (Fc) DyLight680 (ThermoFisher Scientific) and goat anti-human IgA (alpha chain) DyLight800 (Rockland Immunochemicals) antibodies at a final concentration of 0.1 µg/ml and 1 µg/ml, respectively (in 10% RL/PBS-T for 45 minutes at room temperature). Subsequent washing (3 times with PBS-T for 1 minute) was followed by dipping the microarrays in 1mM TRIS pH 7.4 and drying with pressurized air. Images were acquired with a LI-COR Odyssey CLx Infrared Imaging System (scanning offset 0.65 mm, resolution 21 µm). The resulting 32-bit gray-scale TIFF files were converted into 16-bit gray-scale TIFF files using ImageJ (Fiji) software and subsequently analyzed with PepSlide^®^ Analyzer (SICASYS Software GmbH). The PEPperPRINT software algorithm calculated median foreground intensities (background-corrected intensities) of each spot and spot-to-spot deviations of spot duplicates. We tolerated a maximum spot-to-spot deviation of 40%, otherwise, the corresponding intensity value was zeroed. In addition, the microarray scans were reassessed with respect to artifacts by visual inspection, and erroneous values were corrected manually.

### Statistical Analysis

**Figure 1:**
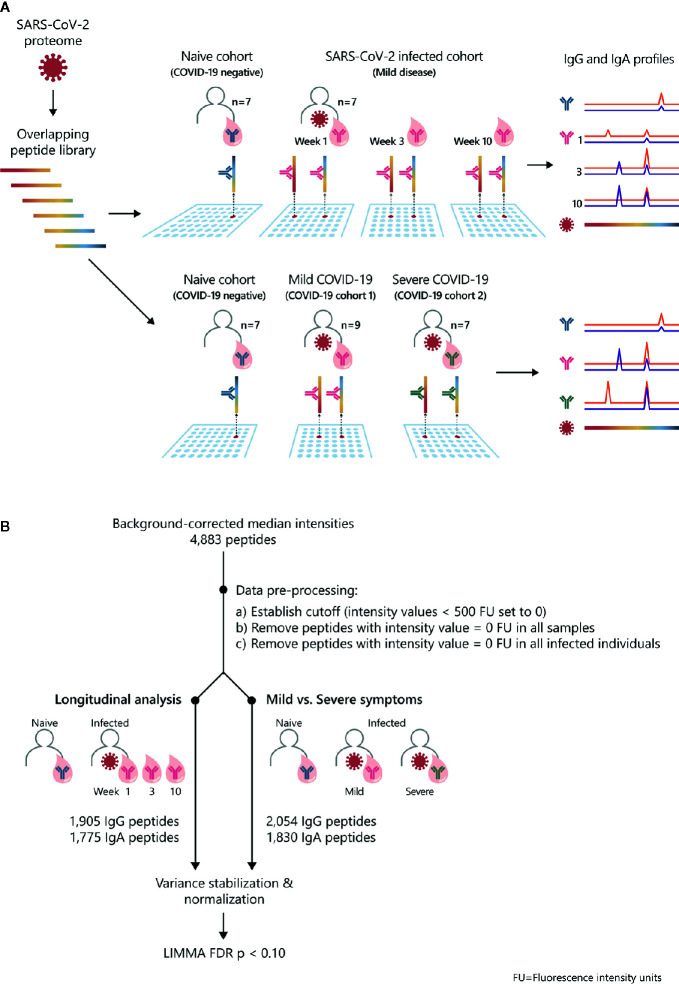
Study overview and statistical analysis workflow: SARS-CoV-2 proteome-wide IgG and IgA epitope mapping. **(A)** The proteome of SARS-CoV-2 was translated in 15-mer overlapping peptides with a peptide-to-peptide overlap of 13 amino acids. The resulting 4,883 individual peptides were printed in duplicates on the microarray. Sera from confirmed COVID-19 patients and SARS-CoV-2-naive individuals were incubated on PEPperCHIP^®^ SARS-CoV-2 Proteome Microarrays. Serum antibody binding was visualized using respective fluorescently labeled secondary antibodies (anti-human IgG and anti-human IgA). Image acquisition and data quantification resulted in epitope-specific antibody profiles for SARS-CoV-2. **(B)** The statistical analysis was performed in the R language (version 4.0.2). Data quantification resulted in background-corrected median fluorescence intensity values (raw data) which were subjected to the following pre-processing steps: (i) signals below 500 FU (fluorescence units) were set as zero, (ii) peptides with 0 FU in all individuals were removed and (iii) peptides with 0 FU in all infected individuals were removed. For the longitudinal analysis, the applied pre-processing steps resulted in 1905 remaining peptides for IgG and 1775 peptides for IgA. For the comparison of patients with mild versus severe symptoms, the applied pre-processing steps resulted in 2054 remaining peptides for IgG and 1830 peptides for IgA. Next, the remaining filtered raw values were normalized using variance stabilizing normalization (VSN) followed by statistical analysis based on the LIMMA algorithm. The false discovery rate (FDR) was controlled at a p-value < 0.1 using Benjamini Hochberg procedure.

The statistical analysis for this study was performed in the R language (version 4.0.2). The detailed statistical workflow is given in **Figure 1B**. The background-corrected median intensities (raw values) of IgG and IgA response values (generated as described in section *Peptide Microarrays*) were used for analysis. A positive response was defined as ≥ 500 FU (fluorescence intensity units) and signals below 500 FU were set to zero, accordingly. Peptides with (i) 0 FU in all individuals and (ii) with 0 FU in all infected individuals were removed from further analysis. For the longitudinal analysis of antibody responses, the applied filtering criteria resulted in 1905 remaining peptides for IgG and 1775 peptides for IgA. For the comparison of patients with mild versus severe symptoms, the applied filtering criteria resulted in 2054 remaining peptides for IgG and 1830 peptides for IgA. The remaining filtered peptide raw values for IgG and IgA isotypes were normalized using variance stabilizing normalization (VSN)  (32). The Linear Models for Microarray Analysis (LIMMA-version 3.44.3) package was used to identify the peptide reactivity for each study in IgG and IgA isotypes by fitting peptide-wise linear models to the normalized data. In the LIMMA package, we used the lmFit and empirical Bayes (eBayes) functions to find the statistically significant peptides between the group comparisons. False discovery rate (FDR) was controlled at the p-value < 0.1 using the Benjamini Hochberg procedure. The respective raw data (background-corrected median intensities) and statistically processed data are summarized in **Supplementary Tables S1**–**S4**.

## Results

### SARS-CoV-2 Peptidome-Wide Antibody Profiling

In order to examine SARS-CoV-2-specific humoral responses on epitope level, we screened peptide microarrays covering the entire SARS-CoV-2 proteome with sera from COVID-19 patients and healthy controls (**Figure 1A**). We investigated antibody profiles (i) longitudinally, in COVID-19 patients (n=7) with mild disease symptoms (28, 29) and (ii) in patients with mild (n=9) versus severe (n=7) COVID-19 disease. We applied a dual isotype read-out, analyzing IgG and IgA specific antibody responses against SARS-CoV-2. Subjects (n=7) with neither anamnestic COVID-19 episode nor detectable SARSCoV-2 S1-antibodies served as healthy control group. The subsequent statistical analysis of the obtained peptide microarray data was performed according to the workflow shown in **Figure 1B**. The data are summarized in **Supplementary Tables S1**–**S4**.

### Longitudinal Monitoring of Epitope-Specific Antibody Responses Across the SARS-CoV-2 Proteome

**Figure 2:**
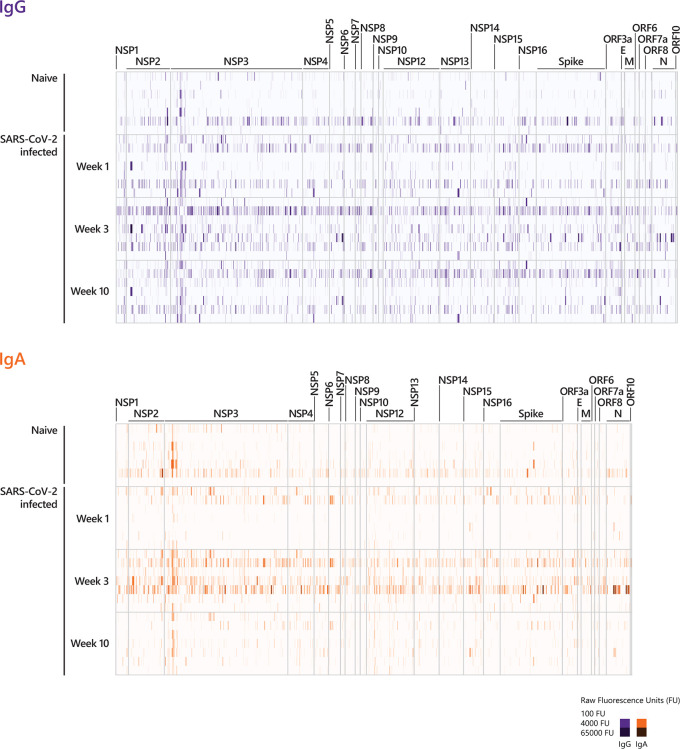
Longitudinal IgG and IgA epitope-specific antibody profiles in COVID-19 patients. For visualization purposes, the heat-maps show the epitope recognition profile for a selected number of peptides (IgG and IgA antibody profiles across the entire proteome are presented in **Supplementary Figure S1**). The selection of peptides was performed applying the following pre-processing criteria: (i) signals below 500 FU (fluorescence intensity units) were set to zero and (ii) peptides with 0 FU in all individuals were removed. This resulted in 2226 remaining peptides for IgG and 2046 remaining peptides for IgA. The heat-maps show the IgG and IgA raw fluorescence intensities for the selected, reduced number of peptides detected for each individual in the corresponding cohort. Sera taken week 1, week 3, and week 10 p.o. from COVID-19 patients with a mild disease course (n=7) and sera taken from SARS-CoV-2-naive individuals were incubated on whole-proteome peptide microarrays. The data for COVID-19 patients for all time points are shown in the following order: patient #2, #4, #3, #8, #7, #1, #10. Serum antibody binding was visualized with fluorescently-labeled secondary antibodies detecting IgG and IgA. The peptide specificities underlying **Figure 2** are provided in **Supplementary Table S5**. NSP, non-structural protein; E, Envelope protein; M, Membrane protein; N, Nucleocapsid Phosphoprotein.

We examined the antibody reactivity in sera of COVID-19 patients collected at different time points post symptom onset (p.o.) (**Figure 1A**). All patients have underwent a mild COVID-19 disease course and recovered (28, 29). Sera from SARS-CoV-2 naive subjects were used as controls. Overall, the breadth of responses was heterogeneous across COVID-19 patients for both IgG and IgA (**Figure 2**, **Supplementary Figure S1**). For IgG, epitope-specific antibody responses were already detectable early after symptom onset (week 1) and increased in most instances towards later time points in both magnitude and breadth. For some epitopes, antibody responses waned over time (**Figure 2**, **Supplementary Figure S2**). Early IgA-specific epitope responses were rather weak but peaked 3 weeks p.o. before declining until around week 10 for most of the epitopes (**Figure 2**, **Supplementary Figures S1**, **S3**). Some background reactivity in the SARS-CoV-2-naive control group was detected, presumably due to cross-reactivity of antibodies against the four endemic human coronaviruses. The heterogeneity of anti-SARS-CoV-2 humoral responses was also shown by ELISA measuring antibodies against the S1 subunit of Spike protein and Nucleocapsid Phosphoprotein and neutralizing antibody titers measured by plaque reduction neutralization test (PRNT) (**Table 1**).

**Table 1:** IgG and IgA serum levels and PRNT50 titers against SARS-CoV-2 of COVID-19 patients and non COVID-19 patients.

patient ID	clinical progress	1 week post symptom onset *			2/3 weeks post symptom onset				9/10 weeks post symptom onset			
		S1 IgG OD ratio	S1 IgA OD ratio	NCP IgG OD ratio	S1 IgG OD ratio	S1 IgA OD ratio	NCP IgG OD ratio	PRNT50^§^	S1 IgG OD ratio	S1 IgA OD ratio	NCP IgG OD ratio	PRNT50^§^
patient 1	mild	0.18	0.34	0.22	1.46	3.04	1.65	>640^#^	1.25	0.94	1.41	160
patient 2	mild	0.22	0.33	0.06	1.56	3.58	0.83	320^#^	3.91	3.82	0.91	80
patient 3	mild	0.24	0.87	0.10	3.26	5.77	0.62	>640^#^	2.38	2.10	0.53	80
patient 4	mild	0.17	0.17	0.18	0.77	2.35	0.43	160^#^	1.24	0.82	0.41	80
patient 7	mild	0.18	0.18	0.30	10.17	>11	6.06	>1280^#^	7.79	>14	5.85	>640
patient 8	mild	0.17	0.15	0.05	3.96	5.94	0.43	>320^#^	5.23	3.59	1.26	320
patient 10	mild	0.26	0.98	0.06	0.28	1.12	0.33	>40^#^	0.87	2.31	1.09	160
patient 14	mild				7.93	9.75	5.30	>40^#^				
patient 16	mild				0.84	1.32		>320^#^				
20-03-CV-09	severe				7.96	>13	4.24	320				
20-03-CV-11	severe				8.1	>12	5.03	>640				
20-03-CV-12	severe				8.14	13.28	6.58	80				
20-03-CV-13	severe				3.49	>9	>9	320				
20-03-CV-16	severe				7.01	>13	4.74	640				
20-03-CV-20	severe				8.26	12.15	6.38	640				
20-03-CV-25	severe				12.13	13.14	6.25	>640				
C01	control	0.16	0.21									
C02	control	0.47	1.07									
C03	control	0.22	0.5									
C04	control	0.24	0.37									
C05	control	0.35	0.25									
C06	control	0.4	0.98									
C07	control	0.3	0.42									

OD, optical density *control sera were obtained from healthy volunteers with no reported symptoms.

^#^Data were previously published by Wölfel et al. (29); ^§^PRNT values are given as reciprocal titers.

**Figure 3:**
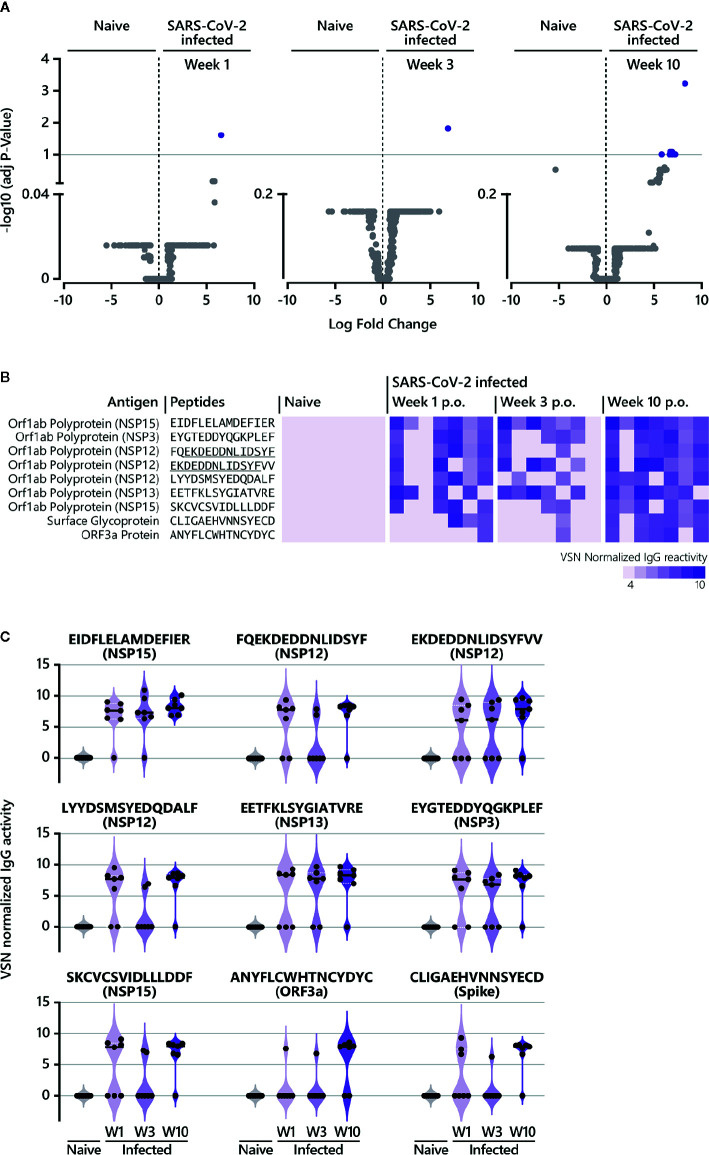
Epitope-specific antibody response in COVID-19 patients over time. **(A)** The LIMMA package was used to determine significant differences in epitope recognition comparing SARS-CoV-2-naive individuals and SARS-CoV-2-infected individuals at different time points p.o. Volcano plots are shown for the comparison between SARS-CoV-2-naive and SARS-CoV-2-infected individuals week 1, week 3, and week 10 p.o., respectively. Volcano plots are depicted with the Log fold change of the intensity of each peptide (x-axis) versus the negative log10 FDR adjusted p-value (y-axis). The Log fold change of a certain peptide is the mean difference in value between the respective groups that are compared. An FDR below < 0.1 was considered as statistically significant. Each dot in the volcano plot represents one peptide. The purple highlighted data points indicate peptides identified for IgG that are significantly recognized in SARS-CoV-2-infected individuals. The grey dots represent peptides that did not reach statistical significance. The results of the statistical analysis are provided in **Supplementary Table S1**. **(B)** Individual IgG antibody responses to the nine peptides significantly identified in COVID-19 patients. The reactivity patterns in all cohorts (naive control group; COVID-19 patients week 1, week 3 and week 10 p.o.) are shown as heat-maps using VSN normalized values. The data for COVID-19 patients for all time points are shown in the following order: patient #2, #4, #3, #8, #7, #1, #10. Peptides underlined indicate overlapping peptides. **(C)** Individual IgG antibody responses to the nine peptides significantly identified in COVID-19 patients as violin plots. p.o., post symptom onset; w, week.

To determine significant SARS-CoV-2-specific epitopes, VSN normalized values from the COVID-19 disease groups were compared to corresponding responses from the SARS-CoV-2-naive group using the LIMMA algorithm (**Figure 1B**, **Supplementary Tables S1** and **S2**, **Figure 3**). For IgG responses, our analysis identified nine peptides reaching statistical significance when COVID-19 patients at week 9/10 p.o. were compared to SARS-CoV-2-negative subjects (**Figure 3A**). Interestingly, antibody reactivity against one epitope (E6662-R6676) was significant across the entire time span of analysis (**Figures 3B, C**). Sequence alignment identified E6662-R6676 as a non-structural protein (NSP) 15-derived peptide. The recognition pattern for the other peptides was rather heterogeneous at the earlier time points, resulting in statistical significance only at week 9/10 p.o. Most of the peptides could be assigned to proteins located in the ORF1a/b polyprotein (**Figure 3B**). The comparison of IgA-specific epitope responses in sera of SARS-CoV-2-infected patients with control sera resulted in no peptides reaching statistical significance. However, when considering peptides solely based on log fold changes (LFC > 4) between naive and infected cohorts, several epitopes were identified (**Supplementary Figure S4B**). Amongst others, the analysis revealed epitopes C649-D663 (Spike protein), A143-C157 (ORF3a protein) and E5466-E5480 (NSP13), which were shared between IgG (**Figure 3B**) and IgA responses (**Supplementary Figure S4B**) at later disease stages.

All identified epitopes with a log fold change > 4 between naive and infected cohorts are summarized in **Supplementary Figure S4**. Our analysis revealed 38 IgG-specific epitopes (excluding the nine peptides described in **Figure 3**) and 16 epitopes specific for IgA together with 10 epitopes that are shared between both antibody isotypes (**Supplementary Figure S4**). For IgG, antibody responses against six epitopes (**Supplementary Figure S4A**) were continuously detectable throughout the time period of analysis as observed for E6662-R6676 (**Figures 3B, C**). Two of the six epitopes (L4534-Y4548 (NSP12), E2940-Y2954 (NSP4)) were also recognized by IgA week 3 and 10 p.o. For most of the peptides identified for IgG, log fold changes > 4 were observed at week 10 p.o. (**Supplementary Figure S4A**), with one peptide shared between IgG and IgA (E2550-L2564 (NSP3)). Measuring IgA-specific antibody responses over time revealed that the majority of identified epitopes peaked week 3 p.o. and waned thereafter (**Supplementary Figures S2**, **S4B**). In contrast to IgG, no IgA-specific epitope was detected at early disease stages (within week 1 p.o.).

In summary, studying the longevity of epitope-specific antibody responses in COVID-19 patients demonstrated the presence of IgG antibodies, which were already detectable quite early during the course of illness until the late convalescent phase in the majority of patients (IgG antibodies recognizing the peptide E6662-R6676 (NSP15)). Apart from that, increasing IgG antibody reactivities were identified over time, with the most robust responses in the convalescent phase such as IgG antibodies recognizing the peptide E5466-E5480 (NSP13). Finally, investigating the dynamics of epitope-specific IgA responses showed a clear peak in the early convalescent phase (week 3 p.o.) before declining for most of the epitopes.

### SARS-CoV-2-Specific Epitope Signature in Patients With Mild And Severe COVID-19 Disease

To identify antibody reactive epitopes associated with mild and/or severe COVID-19 disease, sera from COVID-19 patients taken week 2-3 p.o. and SARS-CoV-2-naive controls were probed on whole-proteome SARS-CoV-2 peptide microarrays (**Figure 1A**). Overall, the breadth of IgG and IgA antibody responses across the proteome varied among individual subjects with more heterogeneous responses in mild cases. However, the range of epitope heterogeneity and response intensities was highly variable within one patient group. (**Supplementary Figure S5**). High variances from undetectable to very strong responses towards Spike S1 and Nucleocapsid proteins were also observed by ELISA, particularly in individuals with mild COVID-19. Furthermore, this heterogeneity was reflected in the neutralizing antibody titers, which ranged from >40 to >1280 (**Table 1**). Because the array screening was performed on linear and not on conformational peptide arrays, the experimental setting did not allow a direct comparison of array data with ELISA and PRNT data. Spike S1 and Nucleocapsid ELISAs and PRNT measure immune responses to presumably conformational epitopes, whereas the array experiments detect linear epitopes only.

**Figure 4:**
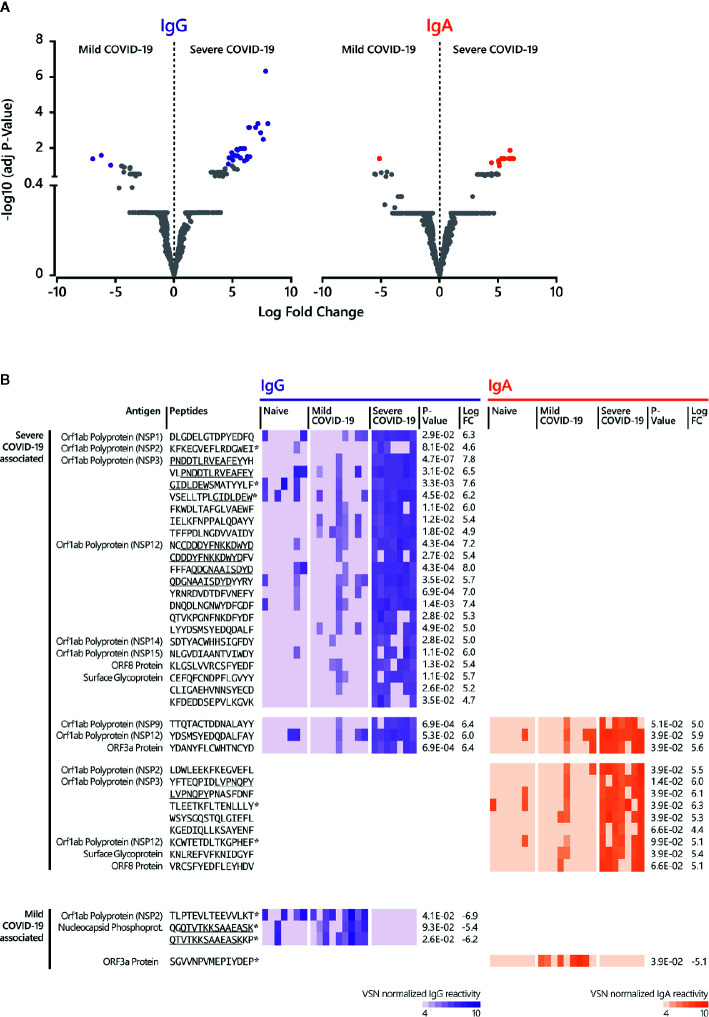
Linear B cell epitopes associated with mild and severe COVID-19 disease. **(A)** The LIMMA package was used to determine significant differences in epitope recognition comparing mild and severe COVID-19 disease. Volcano plots are shown for the comparison between mild and severe COVID-19 disease for IgG and IgA. Volcano plots are depicted with the Log fold change of the intensity of each peptide (x-axis) versus the negative log10 FDR adjusted p-value (y-axis). The Log fold change of a certain peptide is the mean difference in value between the respective groups that are compared. An FDR of < 0.1 was considered as statistically significant. Each dot in the volcano plot represents one peptide. The purple (IgG) and orange (IgA) highlighted data points indicate epitopes that are significantly recognized. For IgG, the right-side purple dots correspond to significant severe disease-specific peptides and the left-side purple dots correspond to significant mild disease-specific peptides. For IgA, the right-side orange dots correspond to significant severe-specific peptides and the left-side orange dot correspond to one significant mild-specific peptide. The grey dots represent peptides that did not reach statistical significance. **(B)** Individual antibody responses to peptides significantly associated with mild and disease severity. The reactivity patterns in all cohorts (naive control group, COVID-19 patients with mild and severe disease) and for both isotypes are shown as heat-maps using VSN normalized values. Underlined peptides indicate overlapping peptides. * indicates no significant discrimination from the naive control group. Adjusted p-value for the comparison between mild and severe disease cohort and Log fold change (LogFC) between mild and severe disease cohort was determined by LIMMA analysis. The results of the statistical analysis are provided in **Supplementary Tables S3** and **S4**.

Further data analysis (**Figure 1B**, **Supplementary Tables S3**, **S4**) revealed significant differential epitopes for mild and severe disease outcomes (**Figure 4**). Compared to mild disease, severe COVID-19 is associated with a higher number of significant, linear B cell epitopes for both IgG and IgA responses (**Figures 4A, B**). Notably, numerous severe-disease epitopes can be assigned to NSP3 and 12 (**Figure 4B**). IgG antibody reactivity against peptide P1622-H1636 (NSP3) was most powerful (adjusted p-value 4.7E-07) to discriminate severe from mild COVID-19 disease, followed by the NSP12-derived peptide N4542-D4556 (p-value 4.3E-04). Although less significant, a NSP3-derived IgA epitope (Y1906-Y1920) was most comprehensive in severe COVID-19 cases (adjusted p-value 1.4E^-02^). Moreover, our approach identified three IgG and IgA shared epitopes recognized in sera from patients with a serious disease course (**Figure 4B**) with the ORF3a-derived peptide Y141-D155 (p-value IgG 6.9E-^04^; p-value IgA 3.9E-^02^) representing the best discriminative shared epitope.

For mild disease, only a few peptides reached statistical significance when compared to the severe cohort (**Figures 4A, B**). When the detected antibody responses against these mild-disease peptides were compared to the naive control group, there was no significant discrimination (adjusted p-value ≥ 0.1).

Taken together, analyzing epitope-specific antibody reactivity in sera from patients with mild and critical COVID-19 clinical presentation identified SARS-CoV-2 specific epitopes which may have the potential to discriminate disease outcome.

## Discussion

A detailed understanding of the immune response following infection with SARS-CoV-2 is invaluable for disease control, identification of new diagnostic markers and development and monitoring of potent antiviral drugs and vaccines. With the aim to study the SARS-CoV-2 specific humoral immune responses and to identify (potential) discriminative markers for disease outcome, we analyzed both the longevity of COVID-19 associated antibodies and the epitope signatures in patients with mild and severe disease. Using peptide microarrays covering the entire proteome of SARS-CoV-2 as overlapping peptides, global IgG and IgA profiles were obtained in patients with mild and severe COVID-19 disease and unexposed healthy controls.

Several studies are available analyzing the time course of antibody responses towards SARS-CoV-2 using cross-sectional and/or longitudinal patient samples (14, 19, 23, 24, 33–42). Most of these studies focus on responses against Nucleocapsid, Spike protein, and subunits of Spike protein, respectively. There is growing evidence that IgM and IgA responses decline rapidly while IgG responses are more stable, potentially waning at later time points and at a slower rate, which is in good agreement with our data. Monitoring longitudinal epitope-specific antibody responses across the SARS-CoV-2 proteome revealed a short-lived IgA and a more stable IgG response. Heterogeneity of individual antibody responses, as observed in our study, is consistent with previous studies (43) with COVID-19 patients not mounting a detectable antibody response to highly variable titers of SARS-CoV-2 specific antibodies.

To our knowledge, there is one study investigating overlapping B cell epitopes across the entire proteome of SARS-CoV-2 in a longitudinal set up (24). By using the same peptide microarray as in the present study, Dahlke et al. demonstrated a strong IgA response early p.o. (week 1) for mild COVID-19 disease, which declined towards week 4. A similar trend was also observed for IgG, however with less reactive peptides as compared to IgA responses (24). This is in contrast to our data, as we detected rather weak early (week 1) IgA-specific responses by both peptide microarray analysis and ELISA. Moreover, IgG responses increased towards week 3 p.o. with a slight decrease towards week 10. The discrepancy between the two studies might be due to the number of samples studied, as the study by Dahlke and co-workers (24) analyzed a very small number of patients (with mild COVID-19 disease represented by sequential samples from one patient).

We identified the NSP15-derived peptide E6662-R6676 as a potential serological marker of acute, early and late convalescent (up to day 60-70 p.o.) of mild COVID-19 disease (**Figure 3**). NSP15 (endoribonuclease) has been discussed as a drug target (44, 45) and recently, amongst other SARS-CoV-2 proteins, was identified as an interferon antagonist functioning in immune suppression (46). There is only limited data available on SARS-CoV-2 NSP15 as a target for humoral responses (25, 26). In a study using a protein array covering ~60% of the SARS-CoV-2 proteome, no significant anti-NSP15 responses in COVID-19 convalescent sera were detected (25). However, protein arrays address conformational rather than linear epitopes. A NSP15-derived epitope was identified by peptide microarray screening, however with no sequence overlap to either E6662-R6676 or to the second identified NSP15-derived peptide S6740-F6754 (**Figure 3B**). The absence of sequence overlaps also applies to the other identified peptides reaching statistical significance in our analysis. This might be due to the contrasting amino acid offsets used for peptide microarray synthesis (26). Wang and colleagues screened 15-mer peptides with an offset of 10 amino acids (26), while the PEPperCHIP^®^ SARS-CoV-2 Proteome Microarray comprises 15-mers with an offset of 2 amino acids. Besides, the number of patients was small in both studies (≤ 10) and patients were of different ethnic origin, which might affect epitope recognition.

Recently, antibody responses against ORF8 and ORF3b were described as serological markers of early and late SARS-CoV-2 infection (35) using the luciferase immunoprecipitation system (LIPS) assay. In our analysis, antibody responses against ORF8 did not reach statistical significance when COVID-19 patients were compared to healthy controls. When considering peptides solely based on log fold changes (LFC >4), few ORF8 protein-derived peptides were identified (**Supplementary Figure S4**) for both IgG and IgA. However, in contrast to the study by Hachim and colleagues using full-length antigen-fusion proteins, we did not observe a maintained antibody response targeting ORF8 at the epitope level in any patients. Additional data on antibody responses towards ORF8 are limited (24–27), especially thoseexamining longitudinal samples. For ORF3b, further data on the antigenicity with respect to eliciting humoral responses have not been published until now. ORF3b uses an alternative start codon (ATG) located within the genomic region of ORF3a (47, 48) and whole-proteome studies to date have not yet considered ORF3b (24–27), which also applies for our study. Hence, additional research is necessary to shed more light on anti-ORF3b (as well as anti-ORF8) associated antibody responses. In the case of the NSP15-derived epitope E6662-R6676 discovered in the present study, its suitability as a serological marker for early and late COVID-19 disease needs to be validated in future studies with larger patient cohorts, including longitudinal samples from COVID-19 patients with different disease severities.

For the late convalescent phase of infection, we moreover identified NSP3 (papain-like protease), 12 (RNA-dependent RNA Polymerase), 13 (Helicase), Spike and ORF3a-derived peptides. NSP3, 12, and 13 are also under investigation as drug targets for COVID-19 (49–52), whereas Spike protein is known to mediate host cell entry (53) and ORF3a protein is described as modulating the host immune response to virus infection, hence having a role in pathogenesis (54, 55). Antibody reactivity against ORF3a has already been detected in SARS-CoV-positive patients in 2003/2004 (56) and Akerström et al. determined that the N-terminal ectodomain of ORF3a induces neutralizing antibodies (57). Of note, the Spike protein-derived epitope C649-D663 discovered in our study is located in the S1 domain, adjacent to the S1/S2 cleavage site [AA681-685, (53)]. C649-D663 is recognized by both IgG (**Figure 3B**) and IgA (**Supplementary Figure S4B**). Interestingly, the sequence partially overlaps with immunodominant epitopes discovered by Yi et al. [S1-65; EHVNNSYECDIPIGAGICAS; (58)] and Farrera-Soler et al. [AA655-672; HVNNSYECDIPIGAGICA; (59)] for convalescent COVID-19. The latter study provided evidence that serum with antibodies recognizing the Spike-derived epitope AA655-672 inhibits cleavage mediated by furin (59). Whether antibodies targeting the epitope C649-D663 are also capable of inhibiting furin-mediated proteolysis of Spike remains to be determined. Other reports investigating linear B cell epitopes on SARS-CoV-2 Spike discovered further immunodominant linear epitopes (24, 26, 27, 60–62). Overall, differences in recognized peptide sequences might be explained by differences among (i) applied screening technologies, (ii) peptide lengths (ranging from 15-25 amino acids) and peptide-peptide overlaps (5-13 amino acids) and (iii) patient cohorts and study design (e.g., time points of serum collection).

The clinical picture of COVID-19 is multifaceted with classifications from asymptomatic, mild, moderate to severe disease (4–6). The identification of correlates of COVID-19 disease severity is crucial for clinical management and intervention strategies. In this respect, hematological and immunological markers are evaluated as prognostic biomarkers (63–65). Aiming to uncover epitopes associated with different disease outcomes, we compared the epitope signatures of patients with mild and severe COVID-19 disease in the early convalescent phase (week 3 p.o.). In general, there are reports demonstrating that antibody titers are higher in patients with severe disease (23, 33, 37, 40, 66, 67), however others show the contrary (68). These studies focused on antibody responses elicited by Spike and/or Nucleocapsid protein. To gain a more comprehensive picture, we evaluated antibody responses across the entire SARS-CoV-2 peptidome.

Our analysis revealed several linear B cell epitopes, which were significantly associated with severe COVID-19 disease (**Figures 4A, B**). The majority of these epitopes is derived from proteins encoded in the Orf1a/b locus locus, with sequences originating from NSP3 and NSP12 as being the most common. In fact, the most significant detected peptides recognized by IgG represent the NSP3-derived peptide P1622-H1636 (p-value 4.7E-^07^) and the NSP12-derived peptide N4542-D4556 (p-value 4.3E^-04^); for IgA, the peptide Y1906-Y1920 originating from NSP3 (p-value 1.4E^-02^). Interestingly, the best discriminative peptide for severe COVID-19 recognized by both antibody isotypes can be assigned to the ORF3a Protein (Y141-D155; p-value IgG 6.9E-^04^; p-value IgA 3.9E^-02^). For mildly ill patients, only a minority of peptides reached statistical significance when compared to severely ill patients. So far, only one study evaluated the association of epitope-specific antibody responses with disease severity (60). Two Spike protein- and one Nucleocapsid protein-derived peptides demonstrated a significantly higher magnitude of antibody responses in severe as compared to mild cases at median 23 days p.o. Of note, higher IgG responses in severe cases were not detected earlier during the course of the disease (60). With regard to a possible use as discriminating biomarkers for disease severity, the detection in an early stage of disease would be crucial. In this context, it would be important to validate our potential discriminative markers with sera from early disease stages. Overall, the clinical significance of identified epitopes needs to be verified.

Our study identified only few linear epitopes derived from SARS-CoV-2 Spike protein. This is in contrast to the ELISA data, showing strong responses to Spike protein particularly in patients with severe COVID-19. However, these results did not translate into high immunoreactivity towards Spike-derived microarray peptides. These findings suggest that the immune response to Spike is predominantly directed against conformational epitopes. Structure analyses characterizing the binding modes of neutralizing SARS-CoV-2 antibodies to SARS-CoV-2 Spike protein and the RBD revealed discontinuous and strictly conformational epitopes (69–73), which cannot be addressed by linear peptide microarray screening.

Given the high similarity of the SARS-CoV-2 proteome to SARS-CoV (47) especially in the NSPs, the cross-reactivity of described epitopes needs further investigations (including samples from recovered SARS-CoV patients). Sequence alignments demonstrated few amino acid substitutions between SARS-CoV and SARS-CoV-2 for some epitopes. Whether or not these changes have an influence on antibody recognition has to be further explored. The Spike-derived peptide C131-Y145 (CEFQFCNDPFLGVYY) identified under the most significant matches for severe COVID-19, harbors the highest number of amino acid substitutions (SARS-CoV sequence: AA128-142; CNFELCDNPFFAVSK) suggesting a SARS-CoV-2 specific antibody response. In this context, identified ORF8-derived epitopes might be of great interest as the amino acid sequence identity between SARS-CoV-2 and SARS-CoV is only 40% (47). Another aspect which needs further evaluation is possible immune cross-reactivity with antibodies elicited after infection(s) with seasonal human coronaviruses (74). We included control samples taken from healthy volunteers, however patient samples with confirmed cases of hCoV-229E, hCoV-NL63, hCoV-OC43 and hCoV-HKU-1, should be used in follow-up studies to get a more detailed picture of possible cross-reactivities. Finally, our peptide microarray study is based on the proteome sequence of the virus isolate Wuhan-Hu-1 (GenBank ID: MN908947.3), and so does not yet incorporate sequence information from other SARS-CoV-2 isolates. Genome-wide analyses of different SARS-CoV-2 isolates revealed sequence variations across the genome and thus also substitutions at the amino acid level (75, 76). It remains to be determined whether the discovered epitopes in our study are conserved across multiple SARS-CoV-2 isolates.

Altogether, our study identified linear B cell epitopes potentially applicable for early and/or late COVID-19 disease detection and as biomarkers able to discriminate severe from mild disease courses.

## Data Availability Statement

The raw data supporting the conclusions of this article will be made available by the authors, without undue reservation.

## Ethics Statement

The studies involving human participants were reviewed and approved by Ethics Committee of Charité Universitätsmedizin Berlin (EA2/066/20, EA1/068/20) and Ethics Committee at the Medical Faculty of the Ludwig Maximilians Universität Munich (vote 20-225 KB). The patients/participants provided their written informed consent to participate in this study.

## Author Contributions

TS, KH, MM, CD, VS, and VC conceived the study. FK, LS, C-MW, and MH were involved in the collection of clinical samples. TS and KH performed the experiments. KH and FC designed the figures. TS, KH, YM, RS, VS, and VC performed analysis and interpretation of data. TS, KH, VS and VC wrote the manuscript. All authors contributed to the article and approved the submitted version.

## Funding

This study was supported by the Clinical Study Center of Berlin Institute of Health, Charité—Universitätsmedizin Berlin, the Berlin University Alliance, and the German Federal Ministry of Education and Research (NaFoUniMedCovid19—COVIM, FKZ: 01KX2021 and PROVID: 01KI20160A). We acknowledge support from the German Research Foundation (DFG) and the Open Access Publication Fund of Charité—Universitätsmedizin Berlin.

## Conflict of Interest

KH, YM, FC, and RS are employees of PEPperPRINT GmbH, Germany, producing high-density peptide arrays and providing screening services. VS is the CEO and cofounder of PEPperPRINT. MM and VC are named together with Euroimmun GmbH on a patent application filed recently regarding the diagnostic of SARS-CoV-2 by antibody testing.

The remaining authors declare that the research was conducted in the absence of any commercial or financial relationships that could be construed as a potential conflict of interest.

## Supplementary Material

Supplementary Figure 1 Longitudinal IgG and IgA epitope-specific antibody profiles in COVID-19 patients across the entire SARS-CoV-2 proteome. The heat-map shows raw fluorescence intensities for each peptide detected for each individual in the corresponding cohort. Intensity values below 100 FU were set to zero. Sera taken week 1, week 3, and week 10 p.o. from COVID-19 patients with a mild disease course (n=7) and sera taken from SARS-CoV-2-naive individuals were incubated on whole-proteome peptide microarrays. The data for COVID-19 patients for all time points are shown in the following order: patient #2, #4, #3, #8, #7, #1, #10. Serum antibody binding was visualized with fluorescently-labeled secondary antibodies detecting IgG and IgA. The peptide specificities underlying **Figure S1** are provided in **Supplementary Tables S1** and **S2**. E = Envelope protein; M = Membrane protein; N = Nucleocapsid Phosphoprotein.

Supplementary Figure 2 Longitudinal IgG epitope-specific antibody profiles in COVID-19 patients presented as percentage responders. For visualization purposes, the longitudinal epitope recognition profile is shown for a selected number of peptides as percentage responders. Peptide selection was performed applying the following pre-processing criteria: (i) signals below 500 FU (fluorescence intensity units) were set to zero, and (ii) peptides with 0 FU in all individuals were removed. This resulted in 2226 remaining peptides. Groups of responders were defined as follows: 0%, 1-20%, 21-40%, 41-60%, 61-80%, 81-100% and color-coded accordingly. The peptide underlying specificities **Figure S2** are provided in **Supplementary Table S5**.

Supplementary Figure 3 Longitudinal IgA epitope-specific antibody profiles in COVID-19 patients presented as percentage responders. For visualization purposes, the longitudinal epitope recognition profile is shown for a selected number of peptides as percentage responders. Peptide selection was performed applying the following pre-processing criteria: (i) signals below 500 FU (fluorescence intensity units) were set to zero and (ii) peptides with 0 FU in all individuals were removed. This resulted in 2046 remaining peptides. Groups of responders were defined as follows: 0%, 1-20%, 21-40%, 41-60%, 61-80%, 81-100% and color-coded accordingly. The peptide underlying specificities **Figure S3** are provided in **Supplementary Table S5**.

Supplementary Figure 4 Longitudinal IgG and IgA epitope-specific antibody reactivity patterns against selected peptides in COVID-19 patients. Individual IgG **(A)** and IgA **(B)** antibody responses to selected epitopes based on Log fold change > 4 between SARS-CoV-2-negative subjects (n=7) and SARS-CoV-2-infected individuals (n=7) week 1, week 3 and week 10 p.o., respectively. The heat-map shows VSN normalized intensity values for each peptide detected for each individual in the corresponding cohort. The data for COVID-19 patients for all time points are shown in the following order: patient #2, #4, #3, #8, #7, #1, #10. The black dots in the last column indicate the time point (week 1, 3, 10) at which the Log fold change between infected and naïve cohort for a given peptide is > 4. Connected dots indicate consecutive time points at which this was observed. LogFC, Log fold change; p.o., post symptom onset.

Supplementary Figure 5 IgG and IgA antibody responses in COVID-19 patients with mild and severe disease outcome across the SARS-CoV-2 proteome. The heat-map shows raw fluorescence intensities for each peptide detected for each individual in the corresponding cohort. Intensity values below 100 FU were set to zero. Sera taken week 2-3 p.o. from COVID-19 patients with a mild disease course (n=9) and COVID-19 patients with a severe disease course (n=7) were incubated on whole-proteome peptide microarrays. Sera from SARS-CoV-2-naive individuals were used as control. Serum antibody binding was visualized with fluorescently-labeled secondary antibodies detecting IgG and IgA. The peptide specificities underlying **Figure S5** are provided in **Supplementary Tables S3** and **S4**. E = Envelope protein; M = Membrane protein; N = Nucleocapsid Phosphoprotein.

Supplementary Table 1 Peptide microarray data – Longitudinal analysis - IgG.

Supplementary Table 2 Peptide microarray data – Longitudinal analysis - IgA.

Supplementary Table 3 Peptide microarray data – Severe vs mild - IgG.

Supplementary Table 4 Peptide microarray data – Severe vs mild - IgA.

Supplementary Table 5 Data for **Figure 2**_**S2**_**S3** – Longitudinal analysis.
