# Supplementary figures and images for: SARS-CoV-2 Proteome-Wide Analysis Revealed Significant Epitope Signatures in COVID-19 Patients
Source: Front Immunol. 2021 Mar 23;12:629185. doi: 10.3389/fimmu.2021.629185 (PMC8021850; doi:10.3389/fimmu.2021.629185)

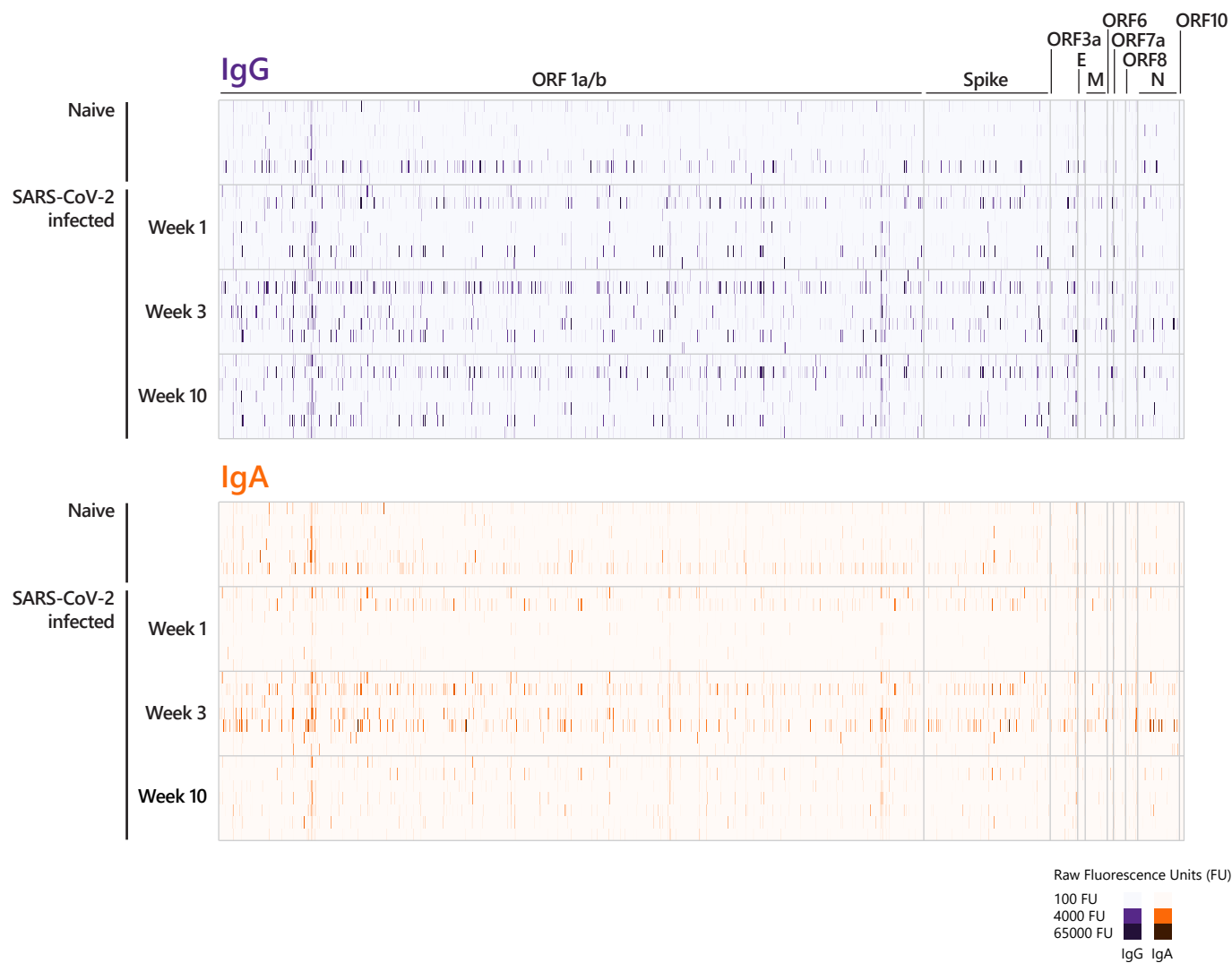

IgG

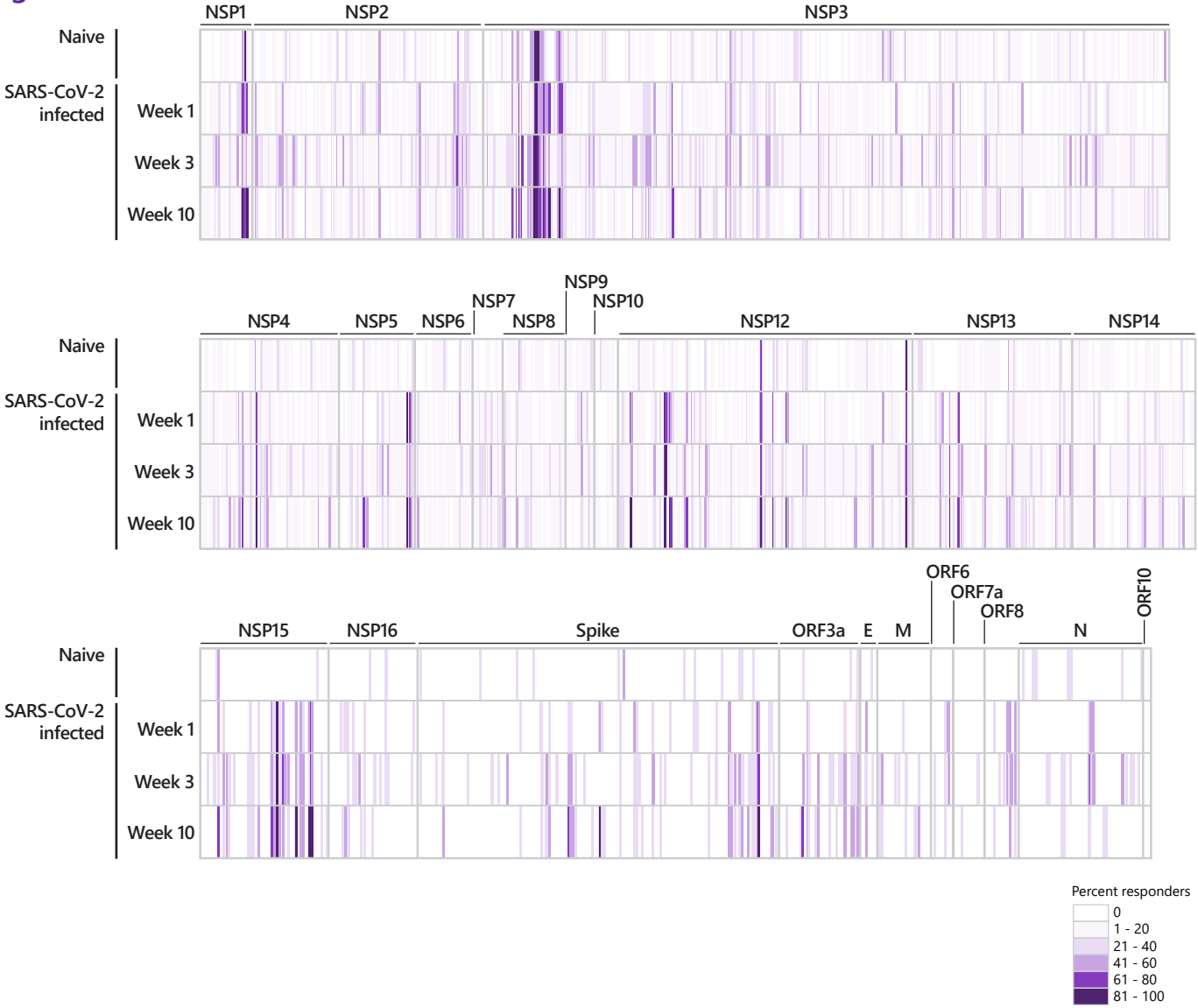

IgA

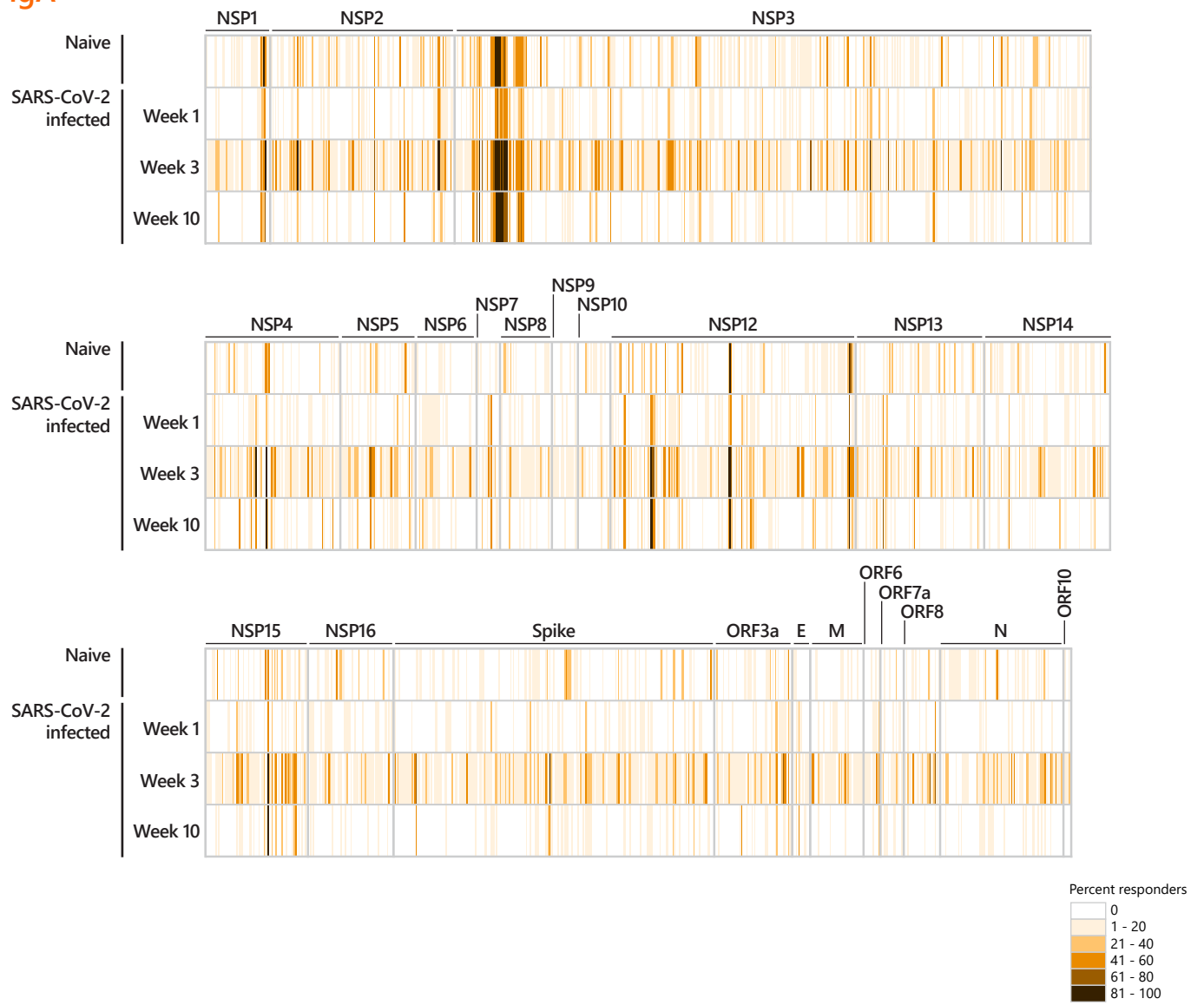

(A)

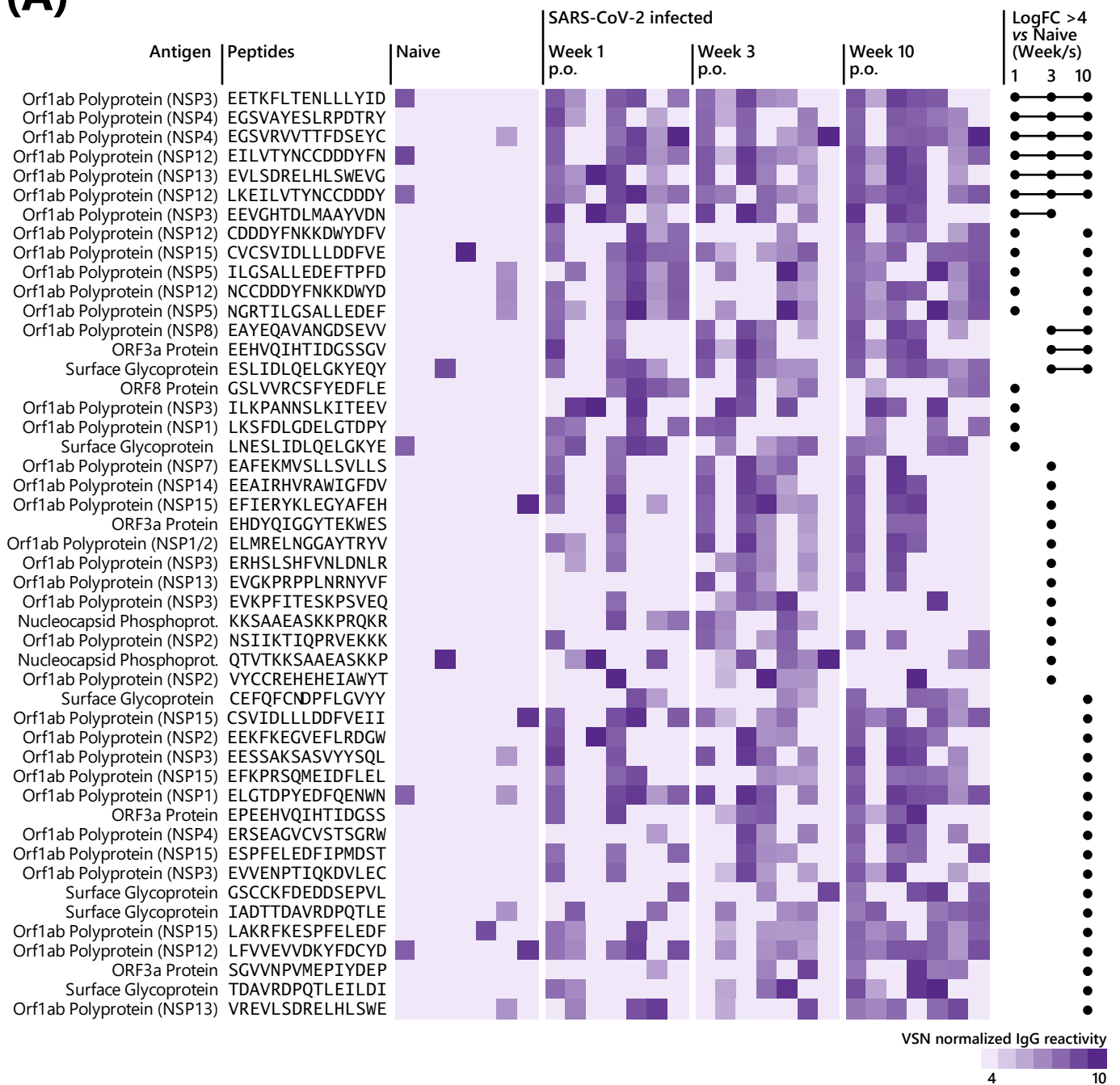

(B)

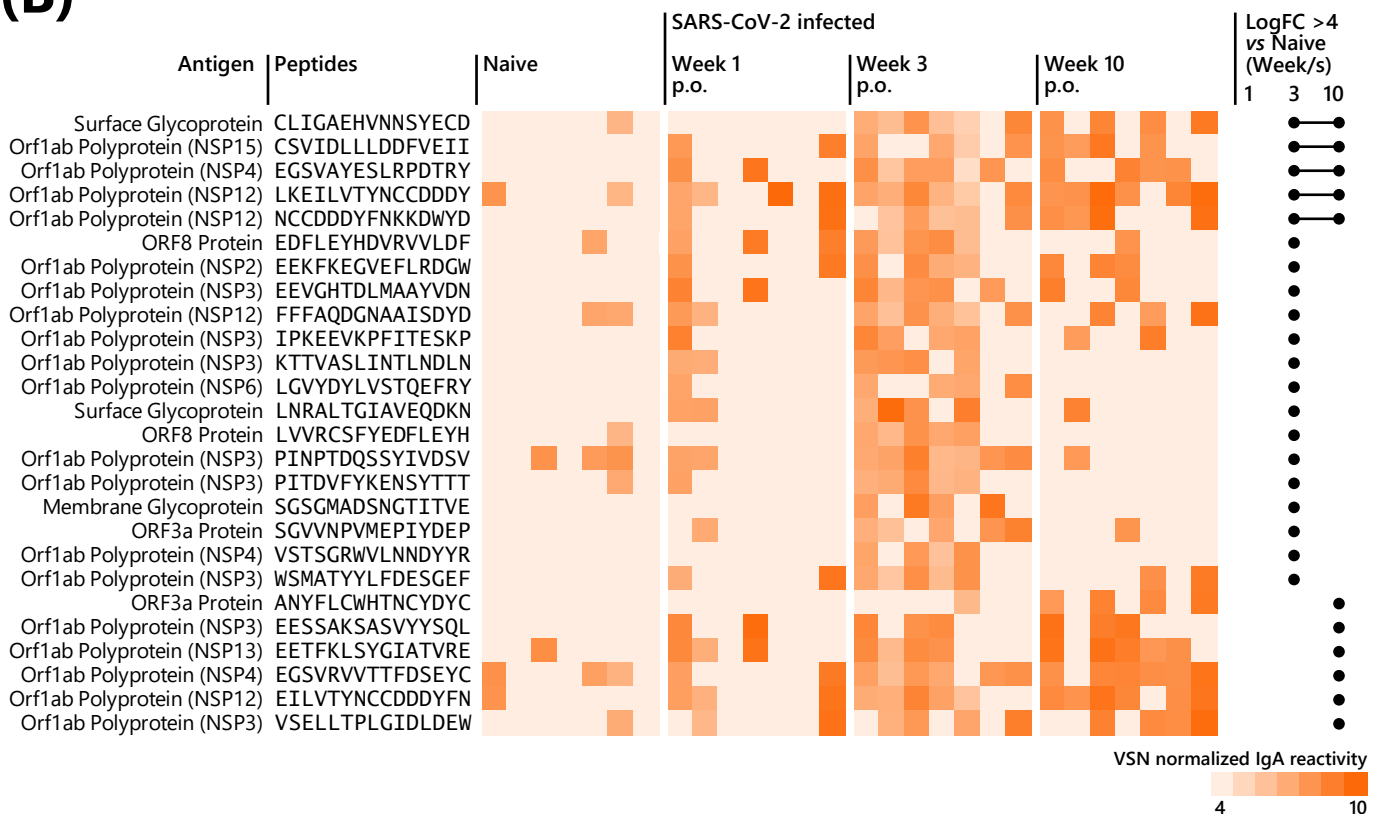

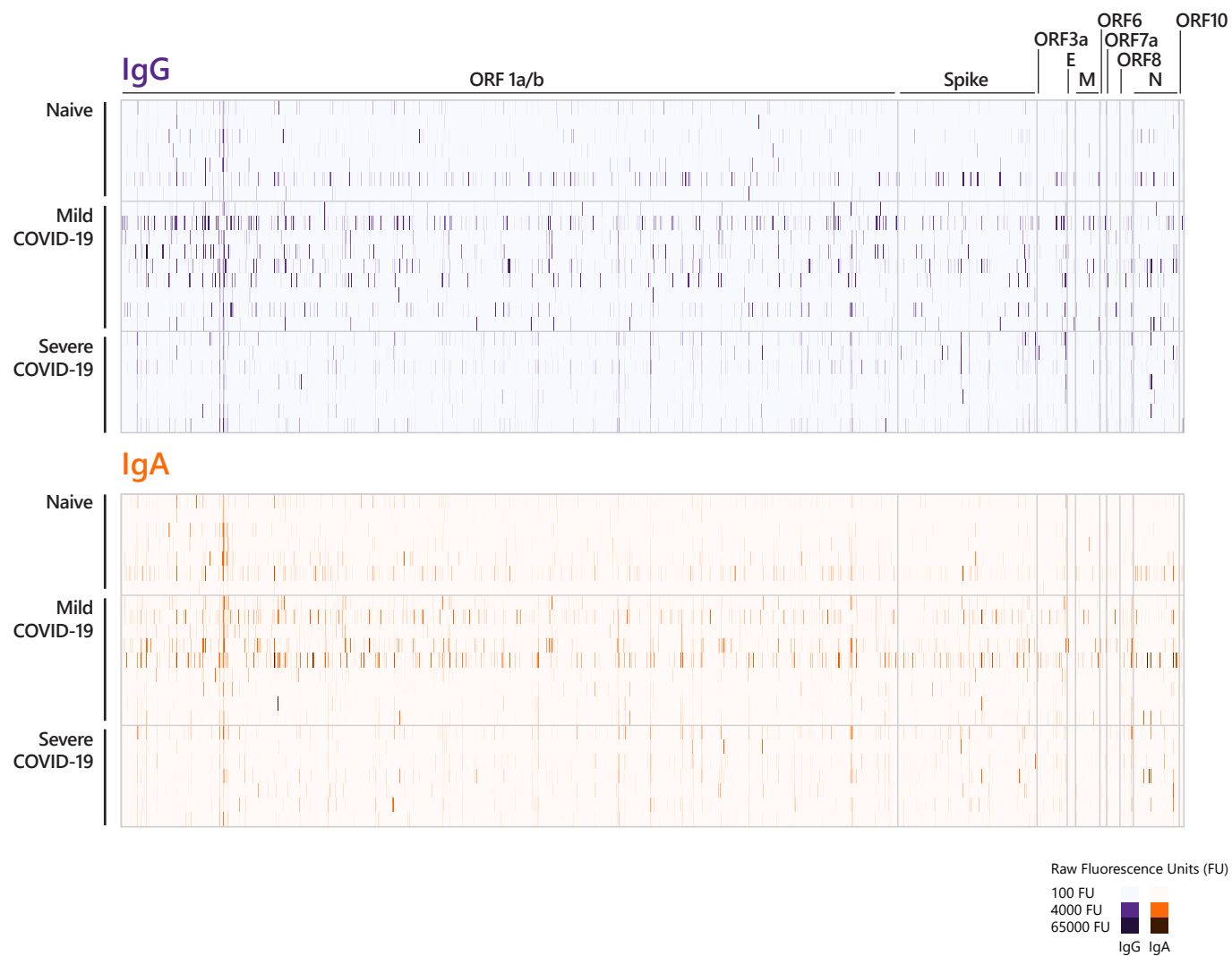

Supplement: Supplementary Figure 1 — Longitudinal IgG and IgA epitope-specific antibody profiles in COVID-19 patients across the entire SARS-CoV-2 proteome. The heat-map shows raw fluorescence intensities for each peptide detected for each individual in the corresponding cohort. Intensity values below 100 FU were set to zero. Sera taken week 1, week 3, and week 10 p.o. from COVID-19 patients with a mild disease course (n=7) and sera taken from SARS-CoV-2-naive individuals were incubated on whole-proteome peptide microarrays. The data for COVID-19 patients for all time points are shown in the following order: patient #2, #4, #3, #8, #7, #1, #10. Serum antibody binding was visualized with fluorescently-labeled secondary antibodies detecting IgG and IgA. The peptide specificities underlying Figure S1 are provided in Supplementary Tables S1 and S2 . E = Envelope protein; M = Membrane protein; N = Nucleocapsid Phosphoprotein. [file DataSheet_1.pdf]
